# Cell-based therapy technology classifications and translational challenges

**DOI:** 10.1098/rstb.2015.0017

**Published:** 2015-10-19

**Authors:** Natalie M. Mount, Stephen J. Ward, Panos Kefalas, Johan Hyllner

**Affiliations:** 1 Cell Therapy Catapult, Guy's Hospital, London SE1 9RT, UK; 2 Division of Biotechnology, IFM, Linköping University, Linköping 581 83, Sweden

**Keywords:** cell therapy, translation, regulation, clinical trial, manufacturing, reimbursement

## Abstract

Cell therapies offer the promise of treating and altering the course of diseases which cannot be addressed adequately by existing pharmaceuticals. Cell therapies are a diverse group across cell types and therapeutic indications and have been an active area of research for many years but are now strongly emerging through translation and towards successful commercial development and patient access. In this article, we present a description of a classification of cell therapies on the basis of their underlying technologies rather than the more commonly used classification by cell type because the regulatory path and manufacturing solutions are often similar within a technology area due to the nature of the methods used. We analyse the progress of new cell therapies towards clinical translation, examine how they are addressing the clinical, regulatory, manufacturing and reimbursement requirements, describe some of the remaining challenges and provide perspectives on how the field may progress for the future.

## 1. Introduction

Cell therapy represents the most recent phase of the biotechnology revolution in medicine. As with many remedies, cell therapies are based on ground-breaking scientific discoveries and technology advancements. Most cell-based therapies are currently experimental, with a few exceptions such as haematopoietic stem cell (HSC) transplantation which is already a well-established treatment for blood related disorders [1,2]. The next generation of cell therapies now emerging are of diverse class. Cell therapies can be classified by the therapeutic indication they aim to address, e.g. neurological, cardiovascular, ophthalmological; by whether they comprise cells taken from and administered to the same individual (autologous) or derived from a donor (allogeneic); or most commonly by the cell types, often using the EU regulatory classification. The EU regulatory classification of cell-based therapies discriminates between minimally manipulated cells for homologous use (transplants or transfusions) and those regulated as medicines which are required to demonstrate quality, safety and efficacy standards to obtain a marketing authorization before becoming commercially available (referred to as Advanced Therapy Medicinal Products; ATMPs) which are further subdivided into somatic cell, gene therapy and tissue engineered products. Another way of considering the diversity of cell therapies is classification by their underlying technology. Broadly, the ATMP subdivisions are mirrored in the cell-therapy technology classification described in this paper. The technology, i.e. methodology, being used, rather than the specific cell type is often the feature that needs to be addressed to solve manufacturing, regulatory and clinical issues in a more general way. Thus, a technology classification can emphasize the commonality in translation challenges between otherwise diverse types of cell-based therapy.

Beyond the diversity of cell therapies and how they are classified, there are common themes in the translational challenges that need to be overcome to bring these therapies through the clinical development process to become available for patients. Recent analyses have shown that the majority of cell-based therapies are still at an early stage of development (clinical trial Phases I and II focused on demonstration of safety and early indication of efficacy) with relatively few reaching the later stages of clinical trial and marketing authorization [3,4]. In addition, it is clear that this field of medicines development is unusual in that, while there is increasing involvement of large pharmaceutical companies and formation of biotech companies, the majority of the clinical trials in this area are still taken forward by academic researchers in universities and hospitals. Experience in the field to date has shown that this is still an emerging area of science and hence cycles of iterative learning are very important, with a close relationship between laboratory researchers and trial physicians to analyse the data from early clinical trials and cycle back to product improvements to build the next generation of therapies. Particular examples of this are in the field of gene-modified T cells where the current generation of anti CD19 chimeric antigen receptor (CAR) T-cell therapies (T cells which are gene-modified to enable antibody-like recognition of the CD19 antigen expressed on B cells) now showing compelling efficacy in B-cell leukaemias have emerged from over 20 years of clinical exploration and cycling back to the laboratory for improvements [5,6].

The types of translational challenge faced in the field, range from the scientific and pre-clinical to those of clinical development. In this article, we focus on the clinical development challenges, ranging from the complexities of designing and running clinical trials with cell-based therapies to how they are regulated and manufactured, and then considering the importance of understanding and early planning of their reimbursement. While these are all rightly described as translational challenges, there are increasing numbers of cell and gene therapies that have successfully navigated the development process, with five ATMPs now approved in the EU. The approved ATMPs include not only cell types which are classified as somatic, including dendritic cells of the immune system (Provenge^®^), cartilage-derived chondrocytes (ChondroCelect^®^ and MACI^®^) and corneal limbal stem cells (Holoclar^®^) but also an *in vivo* gene therapy (Glybera^®^). Additionally, the rapid progress made in the field of *ex vivo* gene modification means an early approval in the gene-modified T-cell class can be anticipated. Taken together, these therapies along with the broad spectrum of other cell therapies earlier in development exemplify how translational challenges can be overcome and how we can apply cycles of learning to accelerate the progression of cell therapies towards commercialization to meet the needs of patients.

## 2. Cell-based therapy technology classification

It is becoming evidently clear that the landscape of cell-therapy development status and use is due to change considerably in the upcoming years driven by very positive efficacy data in the immune cell-therapy field as one recent example [5,6]. These recent data in immune cell-based therapies use viral vector transduction technology to deliver modified genes into T cells to specifically target certain blood cancers. The viral vector technology was originally developed in the 1970s [7] and has been refined over a number of years for various purposes including therapeutic use. Early *in vivo* gene therapies used this technology around the turn of the millennium [8] and now it is being applied further in the cell-therapy field. This is one example of a ground-breaking basic technology that after refinement developed into applications used in the clinic for the benefit of patients. Thus, it might be useful to look at the cell-therapy field from a technology viewpoint rather than from a cell-type perspective, which is the most common approach used. As in the examples above, technologies develop overtime, new methods are added and sometimes technologies become disruptive for an application, such as cell therapy. Increasing the awareness of new technologies in basic science may help to trigger early adoption by translational scientists which could spark the development of new cell therapies.

**Figure 1.**
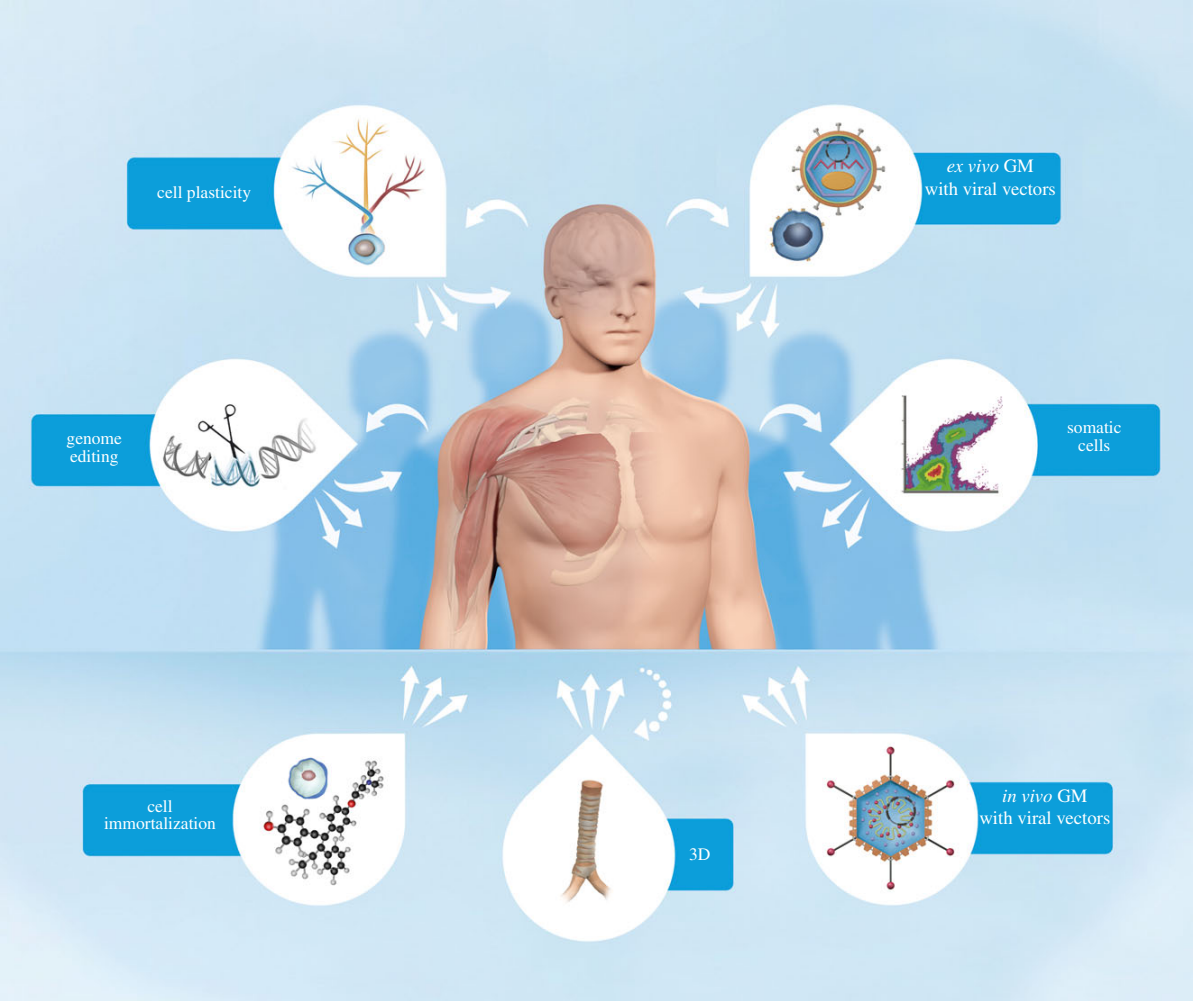
Illustration of cell-technology classification in relation to potential therapeutic use. Key: long arrow towards the human body indicates an autologous approach; short arrows indicate the potential for allogeneic approaches; dashed arrow indicates combinatorial use of cells in 3D technologies; GM stands for gene modifications. The bubbles accompanying each classification graphically illustrate specific technology characteristics as follows: *Ex vivo* GM with viral vectors: a somatic cell and a generic lentivirus enclosing a vector containing a gene sequence of interest; Somatic cells: a flow cytometry diagram, a method often used to purify or characterize somatic cells prior to usage based on cell surface marker expression; *In vivo* GM with viral vectors: a generic adenovirus enclosing a vector containing a gene sequence of interest; 3D technologies: a trachea exemplifying a biological three-dimensional scaffold; Cell immortalization: a generic cell and the molecular structure of 4-hydroxytamoxifen, a compound used as an immortalization regulator; Genome editing: a scissor cutting a DNA strand; Cell plasticity: a pluripotent stem cell differentiation tree symbolizing cell plasticity.

To facilitate an analysis of the various technologies that are being used in the cell-therapy field, it is helpful to classify each methodology into technology areas. The following classifications are introduced for technologies that involve cells in various ways to treat diseases and a brief description of each technology area follows below and are illustrated in figure 1:

— somatic cell technologies
— cell immortalization technologies
— *ex vivo* gene modification of cells using viral vector technologies
— *in vivo* gene modification of cells using viral vector technologies
— genome editing technologies
— cell plasticity technologies
— three-dimensional technologies
— combinations of the above

### (a) Somatic cell technologies

This technology uses cells from the human body that are purified, propagated and/or differentiated to a specific cell product that subsequently is administered to a patient for a specific therapeutic treatment without further technological input. Thus, from a technology viewpoint, the translational challenges are similar despite the heterogeneous cell types that are included in this technology group. Examples of such cells are red blood cells, platelets and chondrocytes and also tissue stem cells such as haematopoietic stem cells (HSC), mesenchymal stem cells (MSC) and skin stem cells, to mention a few. Although the purification, propagation and differentiation methodologies may be very advanced, the general technology innovation factor is normally low. Some treatments using this technology are currently best practice and have been for some time, e.g. blood transfusion and bone marrow transplantation, as these cells were historically easy to access after identification and relatively easy to use for good reasons. Some further cell types are in the clinic and are being used globally, e.g. chondrocytes and skin stem cells [9–11]. MSCs or subpopulations of MSCs are widely popular among translational scientists and several hundred clinical trials are currently ongoing throughout the world [4]. Several trials are in phase II/III or III and potential efficacy data from these large trials could be anticipated to become public within 24 months.

Many other tissue-specific stem cells or progenitor cells may represent an opportunity to become established therapies over the next decade or so. Typically, there are a very small number of stem cells in each tissue and, once removed from the body, their capacity to divide appears to be limited, making generation of large quantities of stem cells difficult [12,13]. These basic challenges need to be addressed before any such therapy can become commercially viable. Recent studies, however, demonstrate that propagation to sufficient quantities may be achievable, at least in some tissue stem cells as reported for human cardiac and liver stem cells [14,15].

A variety of immune cells, such as tumour infiltrating lymphocytes (TILs), viral reconstitution T cells, dendritic cells, *γδ* T cells, regulatory T cells (*T*_reg_) and macrophages are also somatic cells that are being developed as cell therapies. These have a highly specialized mode of action and all these cell types have entered into various stages of clinical development particularly for cancer treatments. Albeit these immune cells fit well within the definition of somatic cell technologies, the translational challenges may sometimes be more complex than normally experienced within this technology area. On the other hand, genetically modified T cells using viral vectors fall under a different technology area because of the modification methodology being used and are therefore described in further detail later.

### (b) Immortalized cell lines

The most well-known example of this technology area is the neural stem cell line CTX [16]. Derived from fetal cortical brain tissue, CTX is a clonal cell line that contains a single copy of the c-mycERTAM transgene delivered by retroviral infection [17]. Under the conditional regulation by 4-hydroxytamoxifen (4-OHT), c-mycERTAM enables large-scale manufacturing of the CTX cells. The cells are currently in clinical phase II trial for stroke. Immortalization technologies have been around some time but are currently not well adopted in the cell-therapy field. If the current clinical trial is successful, an increased attention to this technology area may well be expected.

### (c) *Ex vivo* gene modification of cells using viral vector technologies

*Ex vivo* gene modifications using viral vector technology for cell therapy purposes are used for several types of cells, the most common being T cells [5,6,18], HSCs [19–23] and MSCs [23–25]. Gene modifications of HSCs show promise to treat diseases like ADA SCID (adenosine deaminase severe combined immunodeficiency disease) and gene-modified MSCs are just entering the first clinical trials for indications such as advanced adenocarcinoma. In the case of T cells, which are currently the dominating cell type in this technology field, the approach is to genetically modify the T cells in various ways to target and activate them to effect selective destruction of an assortment of specific cancers. As discussed previously, the research has advanced tremendously during the last few years and many potential therapies have entered into clinical trials [5,6]. It is broadly acknowledged that this research is so promising that it will lead to a paradigm shift within the treatment of haematological malignancies and potentially other areas of cancer medicine in the coming few years [18]. As a consequence, translation of gene-modified T-cell therapies is currently an active area for pharmaceutical companies, who have made large investments during the last couple of years, and the need for increased capacity in GMP (good manufacturing practices) manufacturing of both viral vectors and transduced T cells is a challenging translational area.

### (d) *In vivo* gene modification of cells using viral vector technologies

*In vivo* gene therapy means direct introduction of genetic material into the human body. Although several delivery methods are under development, the most widely used delivery system is to use modified viruses carrying targeting viral vectors that are introduced into human cells via infection *in vivo*. As alluded to in the *ex vivo* gene modification section, the viral vectors most commonly used in ATMPs are retroviral, lentiviral, adenoviral or adeno-associated viral (AAV) vectors [26–30]. Owing to the nature of the viral vector technology, it can be applied to various cell types depending on the intended treatment. Potential indications are numerous and include cancer gene therapy, neurological disorders, (mono)genetic disorders, infectious diseases and cardiovascular abnormalities [27]. The technology area is vast and complex with certain specific translational challenges such as cell targeting specificity and maintenance of controlled expression being among the most significant issues for many therapies in development. This technology is most commonly referred to as gene therapy and is recognized as a specific technology area with great potential in the field of cell-based therapies [26,27].

### (e) Genome editing technologies

Meganucleases, zinc finger nucleases (ZFNs) and transcription activator-like effector nucleases (TALENs) have been used extensively for genome editing in a variety of different cell types and organisms. The greater simplicity of TALENs relative to meganucleases and ZFNs has led to their adoption over the past several years by a broad range of scientists. Lately however, targeted genome editing using CRISPR-Cas9 systems has rapidly gone from being a niche technology to a mainstream method used by many life science researchers because of efficacy and cost reasons reaching a new level of targeting and efficiency [31,32]. Targeted gene editing may still be considered as an evolving and early stage methodology from a translational viewpoint but has the potential to become a disruptive technology within the next decade in the cell-therapy field. Target indications for these gene editing-based therapies will probably start with blood cell related and monogenetic diseases. Autologous HIV treatment by gene editing T cells (CCR5 gene dysfunction) is the first indication to have reached the clinic (finalized phase I) and has used ZFN technology [33].

### (f) Cell plasticity technologies

The cell plasticity technology area takes advantage of discoveries during the last 50 years that certain cells, if not the majority, have the ability to give rise to cell types formerly considered outside their normal repertoire of differentiation. In 1962, John Gurdon removed the nucleus of a fertilized egg cell from a frog and replaced it with the nucleus of a mature cell taken from a tadpole's intestine [34]. This modified egg cell grew into a new frog, showing that the mature cell still contained the genetic information needed to form all types of cells. Similar evidence of cell plasticity was obtained in the 1990s when a mammal, the world famous ‘Dolly the sheep’, was created through nuclear transfer technology [35]. The creation of mouse and human embryonic stem cell lines [36,37] was again a breakthrough, bringing *in vitro* studies of developmental biology and cell plasticity to a new level but also unlocking the door to cellular therapies using this technology. Recently, the field has further evolved in a disruptive manner with the discoveries of mouse and human induced pluripotent stem (IPS) cells [38–40] and the process of transdifferentiation, i.e. the conversion of one differentiated cell type into another, avoiding the pluripotency stage altogether [41–44]. In conclusion, technologies based on cell plasticity hold great promise and clearly have a disruptive clinical potential primarily because of the high probability of an almost unlimited supply of cells and also for the possibility to partly immune match the resulting cell product with the recipient patient [45,46].

### (g) Three-dimensional technologies

Another arm of regenerative medicine, tissue engineering, is combining somatic cell technologies or the varieties of cell-therapy technologies described above, with various types of biocompatible materials to solve structural challenges that are often surgical or immunological in nature. Three-dimensional (3D) technologies, including biomaterial scaffolds, can have many purposes, such as supporting cell viability, induction of cell differentiation, provision of a substrate for cell growth and support for tissue regeneration, provision of the shape, scale and volume of a desired tissue, provision of growth factors and encapsulation of cell transplants to protect the product from the hosts immune system to avoid rejection, to mention a few important examples. In summary, the 3D technologies as a component of a cell therapy can be roughly divided into four subtypes of technologies. These are

— simple biomaterials such as hyaluronic acid, bone substitutes or alginate-encapsulated islets;
— 3D/shaped scaffolds that provide organ shape and bioresorbable substrate for cell growth (e.g. bladder, trachea or 3D printing technologies);
— tissue-derived (decellularized) scaffolds that are 3D but with added benefits of native biomechanical strength and matrix factors such as oesophagus or trachea;
— smart (or second generation) biomaterials that may have thixotropic, thermo-responsive, growth factor-encapsulating or *in situ* self-assembly properties.

The potential for these 3D technologies in therapeutic innovation is very high and multifaceted and readers are referred to excellent reviews [47–51].

### (h) Moving technologies forward

It is beyond the scope of this review to include all the exciting methodologies that are currently under early development in the cell-therapy space. Potentially ground-breaking technologies like self-formation of complex organ buds into organ-like structures, i.e. organoids, is one example of an emerging technology that could become disruptive but is not classified in this paper [52].

The intention with the cell-therapy technology classification is to create a tool to facilitate the development of different therapies using the same underlying technologies in order to create a better understanding of common translational challenges facing these interventions. These challenges, which are further described below, include the manufacturing process, pre-clinical, regulatory and clinical issues and also clinical adoption and health economics.

## 3. Translation into clinical trial

Clinical testing, within the controlled setting of a clinical trial is a critical step to demonstrate the safety and efficacy of a cell therapy. Conducting clinical trials with the classes of cell therapies identified above presents a number of challenges and opportunities, some of which are common across cell-therapy technologies and others which are specific to the cell-therapy type or clinical indication under study. Different cell therapies are currently at different stages in translation (table 1).

**Table 1.** Clinical and manufacturing approaches for cell therapies. The table summarizes the development stage of the cell-therapy technologies with their current manufacturing technologies and key remaining clinical and manufacturing challenges.

cell technology	development stage	remaining clinical challenge(s)	manufacturing technologies	remaining manufacturing challenge(s)
somatic cells	many therapies in phase 2; some reaching later stages	demonstration of compelling efficacy in large randomized controlled studies	manual and automated multi-planar flasks and stack systems; microcarriers within disposable stirred tank systems; hollow fibre growth systems; membrane and contraflow centrifugation systems	scale up and control of large batch sizes. Recovery of cells from microcarriers. Downstream large volume handling, fill finish at scale using enclosed technologies. Suitable potency assays
gene-modified cells (*ex vivo*)	mainly small clinical trials of gene-modified T cells or HSCs; adoptive T-cell therapies reaching large-scale trials	multi-centre trials; treating larger numbers of patients; accelerated development strategy; maximizing efficacy signal while minimizing toxicity	manual processes often not fully enclosed using static bags, gas-permeable pots plus lateral movement bioreactors for higher cell yields. Positive or negative cell selection process steps often used. High cell purity becoming a possibility with smaller footprint sterile cell sorters	adapting systems to deal with variation in quality of incoming patient material. Lack of product stability pressurising manufacturing and distribution models. Lack of real time final product release assays. Low rates of transduction with non-replicating virus. Enclosed and automated solutions are becoming available for the entire process train
gene modification (*in vivo*)	mainly small clinical trials but some proceeding along phase-less accelerated development	consolidation of promising early data into significant long-term efficacy and safety	processes follow a traditional vaccine/biopharma model of upstream (USP) growth of producer cell lines and downstream (DSP) harvesting of replication-defective viral vectors. USP currently limited to manual multi-planar systems but immediate scale-up possibilities exist with commercial automated multi-planar solutions and hollow fibre systems	USP and DSP process scale up currently limiting systemic clinical utility of this technology as yields too low. Step changes needed in USP through scale up adherent systems including microcarriers and disposable dynamic bioreactors. DSP limited by current methodologies so new chromatography and filtration approaches needed for clarification, purification and polishing steps
cell plasticity	mainly pre-clinical with first pluripotent cell-derived therapies reaching clinical trial	demonstration of safety and potential for efficacy in the clinic	current processes are extremely manual, seamless with no intermediate step and rely on small scale culture and harvest technology. High risk processes with QC assays resembling product characterization tests	a bi-phasic process of pluripotent scale up prior to differentiation needed. Intermediate holding step to reduce process risk and increase production options. Dynamic culture systems to expand pluripotent cell numbers. Robotic scale-out of current plate-based technology is also being explored. In process controls deterministic of culture outcomes essential
three-dimensional technologies	mainly pre-clinical tissue engineered therapies with some small-scale trial or clinical case studies	demonstration of safety and potential for efficacy in the clinic	a complex manufacturing interplay between (bio)materials, cells and biological coatings. Incorporates de-cell/recell therapies such as trachea, oesophagus and veins through to smart bandages incorporating cells into an applied external matrix	enclosed bioreactors to control cell and material interface. Improved stability and delivery systems. Robust product to ensure as widespread clinical use as possible

Preparing for and making the transition into an initial clinical trial is a key step for any therapy and for cell-based therapies, the considerations are numerous. Considering the nature of the product and potential risks and benefits, cell-therapy trials start in patients rather than the traditional healthy volunteer route used for small molecules and a seamless development path without the traditional divisions between separate formal phase I (safety), phase II (efficacy detection) and phase III (efficacy and safety confirmation) trials can often follow. For example, Glybera (alipogene tiparvovec), which uses *in vivo* gene modification technology using an AAV vector to replace the gene responsible for the expression of lipoprotein lipase (LPL), was approved in the EU on the basis of clinical data from 27 patients studied in three small non-controlled open-label trials which could be described as combined phase I/II and phase II/III studies [53].

Choosing the right patient population for the initial trial is important and there is a tension between choosing the patients most likely to benefit if the product is efficacious and limiting the risk to which patients are exposed from an experimental therapeutic. An example is replacement retinal pigment epithelial (RPE) cell therapy using cell plasticity technology and cells derived from pluripotent (embryonic or iPS) cells. The loss of the RPE monolayer which supports the neural retina containing the photoreceptors is associated age-related macular degeneration (AMD) [54] and the hypothesis is therefore that replacement of the RPE layer might halt or partially reverse the progression of AMD. However, patients with advanced AMD have been selected for initial trials based on considerations of the risk profile of this novel therapy and due to the physiological course of the illness, patients with advanced disease will have also suffered photoreceptor loss which limits the benefit they might anticipate from a potential restoration of the RPE layer [55].

Cell-therapy trials often require long-term follow-up of trial subjects, to gain important long-term data on both efficacy and safety and follow-up requirements are therefore determined on a case by case basis. A trial of a somatic cell such as an allogeneic MSC may only require limited follow-up for 12 months, for example, as the cells are generally accepted to act in a relatively short-lived immune-modulatory manner. On the other hand, a trial of a technology using cell plasticity for long-term cell replacement or gene modification will require longer term follow-up, perhaps up to at least 15 years depending on the cell type and the risk [56,57].

While the features discussed earlier summarize some of the factors that make cell-therapy trials different to clinical trials of more traditional medicines, it is also the case that many of the principles of good clinical development can apply equally to cell therapies. For example, cell therapies need to demonstrate a compelling efficacy and safety profile to regulators and payers and therefore trials need to be designed appropriately. For example, a trial of an MSC for a cardiovascular indication where the therapy needs to demonstrate benefit over the current standard of care will require a large, statistically powered, randomized, blinded and controlled pivotal trial (e.g. Teva Phase 3 study of mesenchymal precursor cells for chronic heart failure NCT02032004). On the other hand, a gene therapy such as Glybera for a rare indication as described above only required a small development programme to convince regulators of its favourable profile, with payer discussions ongoing [58]. Cell therapies are costly and complex therapeutics and therefore they will be best suited to where they can offer a compellingly large efficacy signal in an indication where there is no suitable alternative therapy or where they can provide a cure rather than symptom or disease management.

The clinical safety risks associated with cell therapies depend on many factors, including their technology type, inherent characteristics such as differentiation status and proliferation capacity, whether the treatment is autologous or allogeneic, whether short-term or long-term cell survival is anticipated, the site and method of implantation and the disease environment into which they are introduced, as well as extrinsic risk factors such as quality control in the manufacturing process. These risks have been reviewed in detail in other publications [59–61] and here we will briefly discuss three main categories of risk that are related to the technology type, namely tumourigenicity, immunogenicity and risks resulting from the cell-implantation procedure.

Tumourigenicity concerns differ between cell technologies. For example, *ex vivo* and *in vivo* gene modification are associated with the risk of insertional mutagenesis [62] through activation, silencing or dysregulation of genes. Early trials reported resulting leukaemias or pre-leukaemias in three gene therapy trials of retrovirally modified HSCs [63]. Our understanding of the risks related to insertional mutagenesis, related to disease background, cell type to be transduced and vector characteristics have now substantially improved and a range of viral vectors are now being successfully used in clinical trials. Therapies using cell plasticity are also considered at a relatively high risk of tumourigenicity, in this case due to the concerns about transfer of remaining pluripotent cells with the differentiated product or genetic abnormalities arising during cell derivation and culture. There are extensive pre-clinical characterization methods now employed to screen for such risk [64,65]. However, it is still early in the translation of therapies derived from pluripotent cells and clinical trials will employ risk mitigation strategies as well as carefully monitoring for tumourigenicity.

Immunogenicity is a challenge to both efficacy and safety as immune rejection of cells will limit their survival and function and adverse immune reactions can result from, or be caused by, transplanted cells. Immunogenicity is influenced by multiple factors including the allelic differences between the product and the patient, the relative immune privilege of the site of administration, the maturation status of the cells, the need for repeat administration and the immune competence of the host. Therapies derived using plasticity technology such as cell re-programming have been shown to have relatively low immunogenicity pre-clinically, as have some somatic cells such as MSCs [66,67] but the pre-clinical situation may not reflect what happens as the cells mature in the patient or following repeated administration and on the whole, allogeneic therapies from across the technology classes require to be administered with immunosuppressants and these are associated with safety concerns, especially if they are required to be maintained over the long term.

The techniques used to implant the cells or adverse events resulting from the cell-therapy mechanism of action once implanted are another important area of risk. Approaches such as 3D technologies for tissue-engineered products in particular often require complex surgical procedures as has been demonstrated for replacement trachea [68]. The safety of the surgical procedure is inherently linked to the safety of the cell therapy itself and both require careful evaluation to support the overall risk : benefit of the therapy. Well-designed and conducted clinical trials are challenging to conduct for complex 3D tissue replacement products but these are required to demonstrate the potential of these therapies and progress towards licensing such that a well-defined and tested product can be made available to patients.

A critical feature of all clinical trials of cell-based therapies is the importance of the close inter-relationship with manufacturing and logistics. Therapies from across the diverse technology classes are often autologous, requiring cells to be harvested from the patient, received at a manufacturing site and then returned to the patient for re-infusion following manipulation. Physicians and triallists, therefore, need to work in close coordination regarding logistical scheduling and patient management during this period and the health and concomitant medication of the patient will impact both the successful manufacture of a suitable quality cell product and achievement of the trial endpoints. It is for this reason that many early trials of cell-based therapies include feasibility, examining successful manufacture and subsequent dosing, as an endpoint. Allogeneic therapies, on the other hand, are more frequently able to employ cell banking and hence large-scale manufacturing batches, but the end product still requires some final preparation which introduces the requirements for logistical coordination and specialist handling at the clinical site. Manufacturing innovation, both in production and supply chain, will be critical to the successful large-scale trial and subsequent rollout of cell base therapies, as will continuing evolution of regulatory requirements as well as infrastructure development with the health system.

## 4. Manufacturing development; no longer hidden in the shadows

The more complex the therapeutic agent, the more important a sound manufacturing strategy becomes. The challenges inherent in translating a research grade method to a reproducible and robust manufacturing process suitable for routine production are extremely significant. As the sector has matured, the appreciation of the size and complexity of the challenges to be overcome have gradually been accepted. However, there is still generally an under-investment in manufacturing development activities in the translational chain. This is often understandable as without a proved clinical effect, the risk to investment is high. The acceleration through clinical development without investment in underlying manufacturing processes (often termed ‘fail-quickly fail-cheaply’) is not unique to the cell-therapy industry and has ported across from the closely-related biopharma industry, which develops recombinant proteins and monoclonal antibodies. The major difference, of course, is that the developers of classic biological molecules have platform processes that have been developed over the last 20 years to use as an advanced ‘base camp’ from which to launch any future manufacturing process. These platforms are often suitable to make material of sufficient quality and potency for early clinical trials, relatively cheaply and quickly, starting with a common starting population of cells within the working cell bank (WCB). This is in contrast to the cell-therapy industry, which has significant heterogeneity in product technology and the production model. For certain product types, most notably somatic cell technologies like MSCs and *ex vivo* gene-modified CD19+ve T cells, common production methods and approaches are being used; although they remain very broad in their technicality, so could not be labelled true ‘platforms’ at this point in time.

The sector has been traditionally divided into autologous and allogeneic therapies which are often then served by a de-centralized or a centralized production model, respectively. This distinction, however, has started to become eroded as company and health-provider strategies evolve, with more hybrid models emerging. A key driver to determining the production model has been product stability, with autologous products often having short shelf lives of only a few hours necessitating production close to the clinical setting; which is often symptomatic of how these products were developed from within the clinical academic community, as detailed earlier. As the industry develops, this de-centralized production model is going to be the one of clinical/patient choice for certain autologous therapies, especially ones which do not require a high level of manufacturing technology; although high investment costs, along with regulatory challenges of multi-site manufacturing process comparability, are barriers to this model. Others which can benefit from the cost-efficiencies of a centralized model will become a reality when product stabilities are improved, so the current geographical and logistical limitations are removed.

### (a) A cost-based approach to product development

The approach traditionally used by the vast majority of cell-therapy developers has been to allow current process and technology solutions to determine the manufacturing strategy. This can often be seen as an attractive option, as superficially it allows a relatively quick and low-risk path to production. This strategy, however, has many serious and indeed potentially catastrophic flaws when considering the path to commercialization. By ignoring the issues of scalability, automation, raw material supply, intermediate and product stability, grade of clean room, process control and general process robustness/failure rate, at best these process ‘landmines’ will have to be dealt with later delaying clinical update and reducing programme value and at worst, the cost of goods (COGs) could well be in conflict with the price-point acceptable to the payer. For cell therapy, where the cost of goods can be relatively high, costs need to be considered early in the development pipeline.

COGs analysis has been routinely used to help define the decision-making process during drug development. Traditionally, this exercise consists of a detailed analysis of the raw materials, consumables, labour and capital costs. Determining accurate numbers for this analysis can be challenging, especially when used earlier in the development timeline when platform processes are not routinely used, as is the case with cell-therapy manufacture.

A well-referenced example of how an early process was taken through to a commercial setting is that of Provenge^®^ (sipuleucel-T), which is an autologous non-gene-modified dendritic cell immunotherapy indicated for the treatment of asymptomatic or minimally symptomatic metastatic hormone-refractory prostate cancer. In this case, a manual, non-enclosed process with short stability for both the starting material and the final product was used to drive the commercial manufacturing model; the final product has a shelf life of only 18 h. This necessitated the setting up of several very large (160–180 000 ft^2^) manufacturing plants to cover the US market alone and an associated complex and time-critical supply chain network. The magnitude of this operation drove Dendreon to require a high price for the treatment to cover the high COGs [69]. At time of writing, Dendreon, who went into Chapter 11 administration in November 2014, were in the process of being acquired by Valeant ($VRX). One can cite other factors which have had a negative impact on the company, but it is widely recognized that the high COGs of a patient-specific dose of Provenge^®^ should not be repeated if cell therapy is to have a true commercial future.

**Figure 2.**
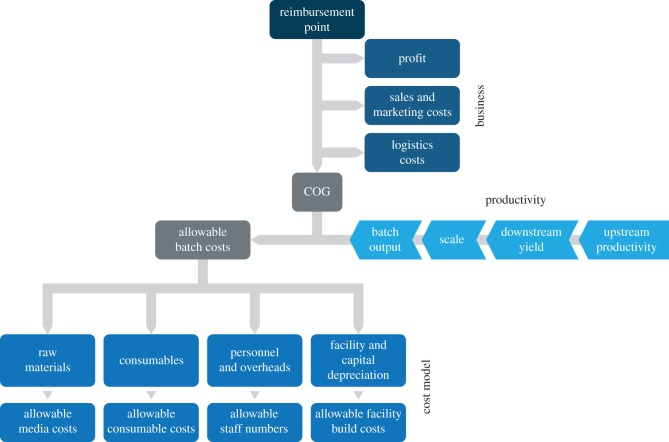
Cost-based manufacturing development model. The reimbursement point is the keystone from which an allowable COGs is determined by subtracting business costs. Manufacturing cost models and associated production technology options can then be systematically investigated to deliver suitable productivity at an allowable batch cost, compatible with the reimbursement strategy.

An alternative to this bottom-up approach is to work back from the price-point that will be acceptable to the payers, to determine the COGs that will be commercially viable. Once this point has been established, a technical development strategy can then be determined to deliver a product at a commercially viable cost. This reverse engineering of COGs allows gross decisions to be made based on the impact that various process options will have on both fixed and variable costs of sales. For example, whether a de-centralized manufacturing strategy can be affordable; what yield per input cost is required, bioreactor selection and downstream processing options; all of which steer the developer to where they should be targeting their development efforts for maximum results. Using this approach, an allowable COGs is determined by subtracting a profit margin, sales, marketing and logistics costs to determine an allowable batch cost. Finally, the batch cost can then be distributed through raw materials, consumables, personnel, overheads and the facility capital costs (figure 2). In this way many different scenarios can be evaluated and the impact on the costs compared. Once the preferred strategy has been identified, a more detailed cost of goods analysis can be performed, to prioritize the process development options.

### (b) Design for manufacture

**Figure 3.**
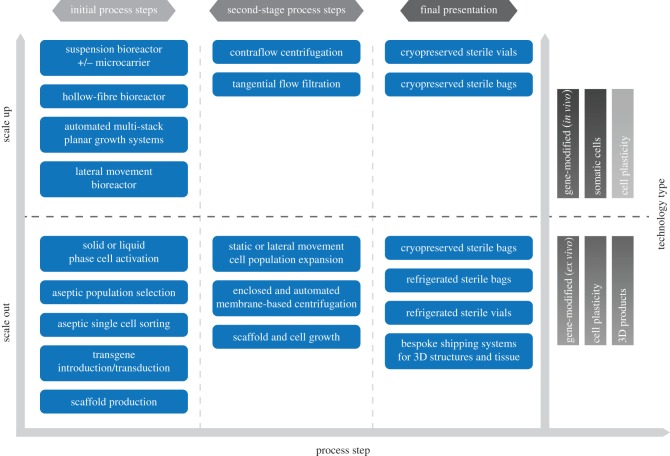
Manufacturing process choices for scale out and scale up of cell technologies. Both the scale of production and the cell-technology type have a significant impact on the production processes used to generate the product. Small scale, patient-specific therapies are commercialized by scaling-out the same process. By contrast, allogeneic therapies are amendable to scale up, which can deliver many identical doses at larger production volumes. Some cell technologies, such as cell plasticity are currently transitioning from small scale-out systems to larger volume scale up production methods.

Working back from the reimbursement price-point will deliver process decisions at the macro level, however, developing a GMP compliant process with associated in process and release assays is a considerable undertaking. Skill-sets, mind-sets, methodology and equipment are different to those required to get to a first in man process, and requires suitable investment. An overview of some currently available manufacturing technologies and their applicable scale is shown in figure 3.

The manufacturing process is often viewed as a series of unit operations performed under GMP conditions at a designated production facility. Product is then shipped to the clinic by a third party specialist, integrator courier or health professional if produced within a local hospital setting. This view may recognize the inter-connectiveness of the steps performed in the ‘factory’, but critically fails to understand the relationship across the entire supply chain. This supply chain includes the impact on product quality because of variation in patient samples, raw materials, interplay between key process parameters and impact of patient delivery at the end of the chain. So, the ‘factory’, which is often seen as the heart of the process clearly has an important role to play, but a significant amount of critical production activity occurs outside of the manufacturing clean room. Understanding this ‘end to end supply chain’ requires a methodological approach, to cut through the myriad of potential factors and issues which could have an impact on product quality, safety and potency, to find the important ones.

The approach which has gained traction in this area is the Quality by Design (QbD) methodologies. At the core of this approach is the identification, evaluation and control of risk [70]. The first steps of the QbD method are for the developer to use risk-based tools to assess the potential risks associated with a unit operation or process step, to categorize the risk and assess the impact of failure. These ‘thought-experiments’ allow a whole range of process preconceptions to be identified and challenged on paper, to determine if they could be potential critical process parameters (CPPs) that have a significant impact on manufacturing; for example, culture seeding densities, feed strategies and harvest shear force. The next step is to determine experimentally if these parameters actually are CPPs, having an impact on the Critical Quality Attributes (CQAs) of the product that are essential to maintain clinical efficacy and safety; such as viability, cell purity and functionality. Understanding the inter-relationship between the CPPs is key, to create an operational space that is understood. For example, to understand the impact of supplier raw material variances to set acceptance tests; to put in place suitable in process controls (IPCs) based on CQAs or their surrogates; and to understand what impact these upstream variables can have on the downstream, such as long-term or near-patient stability.

### (c) Industry step changes needed

To secure a sustainable commercial future, cell-therapy processes need to become more robust, to allow manufacturing to be performed at more than one site and/or geographical location; more reproducible, so batch failure rates are reduced; and as already mentioned, more cost efficient. Automation can have a major positive impact on all three of these challenges. Automation can cover repetitive automation, with a robot mimicking a manual step in a more efficient and reproducible manner (several international vendors for example SelecT from TAP Biosystems, UK; bespoke solutions from Invetech, Australia); bioreactors growing cells in a reproducible manner in suspension or as adherent cultures; cell purification and harvesting systems; and automated fill finish systems (table 1). An often essential component of these automation systems is the dependency on single-use fluid paths, which have the significant advantage of not requiring extensive clean- and sterilize-in place support systems along with the associated validation packages, to ensure process lines are sterilized between batches. The downside to these single-use systems is the upfront work needed to show that the fluid-path polymers have no adverse effect on the therapy. The number of vendors supplying these disposable systems has grown dramatically over the last 5 years, including the level of supporting data that they can provide concerning leachable and extractable molecules from tubing sets and bag films, potentially allowing the entire cell-therapy manufacturing industry to move to use of production rooms which have minimal fixed infrastructure, especially when compared with the classic biopharmaceutical process involving stainless steel bioreactors.

The combination of process automation and single-use fluid paths allows processes to have the potential to be fully enclosed. This can bring significant advantages, not least in operating costs, as the grade of clean room air required for production can be reduced. For example, if a process is aseptic in its nature, as the majority of cell-therapy processes are, and has operational steps that are classed as open, i.e. open to the surrounding environment, then that environment has to be of low particle and low-microbiological burden, as specified in EN/ISO 14644-1 ISO4.8. Enclosing the process to reduce the contamination risk will allow the grade of background environment to be reduced, possibly to ISO 8 or below, depending on the process risk of failure, CMC data package and validation data [71]. While more work is needed to be executed up front, the operating cost savings in clean room build specification, energy and operating costs and environmental monitoring are significant; possibly as high as 50%. Another significant advantage of process enclosure is of course a decrease in the risk of process failure due to contamination. This can be monetized as in the cost of a lost batch but perhaps more importantly, this could be the difference in a patient receiving their autologous treatment or not at all.

To develop an automated process, one still needs to follow the design for manufacture principles described above but once completed the system should deliver the product time after time to the desired specification. To ensure processes which often run into weeks and months are going to deliver the desired product, suitable IPCs need to be developed and qualified to control the process and ensure the correct product will be produced. The plethora of potential parameters which could be used as surrogates of cell quality to define batch outcomes is daunting. In one example, soluble signalling molecules secreted by haematopoietic CD34+ cells have been identified, which generate feedback loops which can be controlled to determine production outcomes [72]. Other potential approaches which can be explored are the identification of patterns within the miRNA (micro RNA) and exosome pools secreted into the culture media by the cells, along with non-invasive imaging which can be quantitated [73]. It is likely that not a single surrogate parameter is going to be sufficient to control the process and all of the above is going to rely on data mining and pattern recognition tools not only to identify the relationships but also to actively control outcomes.

### (d) The significant impact of increasing production yield

A step change in productivity per unit cost needs to be made for the cell-therapy sector. Broadly, there are two approaches to yield increase. The more straightforward option is to produce the desired cells more cheaply. This is the path many are currently following, using the tools, technologies and approaches discussed earlier. An alternative option is to not produce more of the same cells, but to produce cells which have an increase in functionality per unit cost. This can be achieved by different methods depending on the product type, but the underlying principle is that potency per cell dose is as high as possible, allowing fewer cells to be given per dose. Broad approaches to increase yield include *ex vivo* gene modification of cells, increasing the number of desired cells per population through positive or negative selection or representing the tissue niche within the bioreactor to produce cells which are better adapted to survive and elicit efficacious responses within the patient.

## 5. Regulation

Regulation of cell-based therapies in the EU and US follows an established framework broadly divided between minimally manipulated cell therapies for homologous use, which are regulated as tissues or transplants, and more substantially manipulated products, which are regulated as medicines.

The particular features of cell-based therapies, their manufacture and clinical application make the transfer of standards and procedures established for small molecules or biologics challenging, and regulatory requirements have been adapting both to the special characteristics of these products and to respond to patient needs. It is increasingly recognized by developers and regulators that a dialogue is required to navigate and optimize the regulatory system. For example, in the EU, more than minimally manipulated therapies are regulated as ATMPs under a specialized body of the EMA, the Committee for Advanced Therapies, and in accordance with this, scientific guidelines, points to consider and reflection papers have been issued by the EMA on a range of topics as summarized in table 2. Within the EU, approval of clinical trial applications for ATMPs is a national competence, whereas marketing authorizations for ATMPs are only possible through a centralized EU procedure. Scientific advice meetings are a very important part of the development process and available with regulators both during the preparation phase for clinical trial and later in the development process at both national agency and EMA level [74,75].

**Table 2.** Key EU (EMA) regulatory guidance documents and reflection papers for ATMPs. The EMA and its specialist group the Committee for Advanced Therapies publishes guidance documents and reflection papers to assist developers of cell and gene therapies. The table provides their titles and document identifiers.

guidance
— guideline on human cell-based medicinal products (EMEA/CHMP/410869/2006)
— guideline on the non-clinical studies required before first clinical use of gene therapy medicinal products (EMEA/CHMP/GTWP/125459/2006)
— guideline on quality, non-clinical and clinical aspects of medicinal products containing genetically modified cells (EMA/CAT/GTWP/671639/2008)
— guideline on the risk-based approach according to annex I, part IV of Directive 2001/83/EC applied to advanced therapy medicinal products (EMA/CAT/CPWP/686637/2011)
— guideline on safety and efficacy follow-up—risk management of advanced therapy medicinal products [EMEA/149995/2008]
— guideline on scientific requirements for the environmental risk assessment of gene therapy medicinal products [EMEA/CHMP/GTWP/125491/2006]
— detailed guidelines on good clinical practice specific to advanced therapy medicinal products [ENTR/F/2/SF/dn D(2009) 35810]
— reflection paper on stem cell-based medicinal products [EMA/CAT/571134/2009]
— reflection paper on classification of advanced therapy medicinal products [EMA/CAT/600280/2010]
— draft reflection paper on clinical aspects related to tissue engineered products [EMA/CAT/CPWP/573420/2009]
— reflection paper on management of clinical risks deriving from insertional mutagenesis [EMA/CAT/190186/2012]
— European Directorate for the Quality of Medicines—guide to the quality and safety of tissues and cells for human application 1st edition
— Ph. Eur. Monograph 5.2.12 on raw materials for the production of cell-based and gene therapy products [Pharmeuropa—Issue 26.4, 2014]
— annex 2 of Directive 2003/94/EC: manufacture of biological medicinal products for human use
— guideline on potency testing of cell-based immunotherapy medicinal products for the treatment of cancer (CHMP/BWP/271475/06)
— guideline on development and manufacture of lentiviral vectors (CPMP/BWP/2458/03)
— EMA Scientific Guideline: quality, pre-clinical and clinical aspects of gene transfer medicinal products (CHMP/GTWP/234523/09)
— EMA Scientific Guideline: gene therapy product quality aspects in the production of vectors and genetically modified somatic cells (3AB6A)

The novel features of cell-based therapies and their potential to treat diseases which cannot be addressed adequately with current medicines have led to their incorporation into accelerated approvals systems and schemes for access to unlicensed medicines. Examples of this are the new system for the regulation of regenerative medicines in Japan which came into force in November 2014 [76], breakthrough therapy designation and accelerated development path in the USA [77] and the new adaptive pathways scheme in the EU [78]. These paths are being applied to a number of cell-based therapies from the different technology classes. For example, in the EU, a conditional marketing authorization, which is granted to a medicinal product that fulfils an unmet medical need when the benefit to public health of immediate availability outweighs the risk inherent in the fact that additional data are still required, was granted to the Holoclar corneal epithelial limbal stem cell product for the treatment of moderate to severe limbal stem cell deficiency in February 2015. Additionally, an approval under exceptional circumstances in the EU on the basis of only 27 patients in open-label clinical trials was granted in 2012 to Glybera (alipogene tiparvovec) gene therapy for LPL deficiency, a potentially life-threatening, orphan metabolic disease. In addition to accelerated licensing schemes, national agencies can also operate under the EU framework to enable unlicensed medicines to become available to meet the special needs of patients, following the request and under the responsibility of their physician. An additional example of accelerated access initiatives is in the UK where an early access to medicines scheme has been introduced (https://www.gov.uk/apply-for-the-early-access-to-medicines-scheme-eams) and for which a dendritic cell-based approach for glioblastoma was the first to be awarded the new promising innovative medicine designation in 2014.

There are still areas of regulation of cell-based therapies, however, which present challenges for developers, and the majority of these are within the area of quality and manufacturing requirements.

One significant challenge is that raw materials of biological origin are frequently required in the manufacture of cell therapies and sourcing materials of adequate quality can be challenging, with a risk-based methodology increasingly adopted and guidance becoming available (table 2).

Another common challenge with autologous cell therapies in particular is variability of the starting material from the patient and the limited amount of cells or tissue which can be made available for destructive in-process, final release and stability testing. The approval of marketing authorizations for four autologous therapies in the EU to date shows that these challenges can be addressed. In this regard, it is important to define the acceptable variability of starting material, which may have a broad range of acceptability separate to the acceptable variability of the manufacturing process itself, which will usually have a more narrow range. It is the control and variability of the manufacturing process itself and the results of product characterization and release testing that facilitate a robust comparability strategy enabling the effects of changes to the manufacturing process or introduction of a new manufacturing site to be assessed without the requirement for costly clinical bridging studies.

As discussed above, developers are increasingly considering GMP compliant cell manufacturing and characterization at an earlier stage in development and working towards common standards to enable an acceleration towards marketing authorization. An example of this is within the cell plasticity technology area, particularly the induced pluripotent cell space where GMP grade banks and alliances on characterization are emerging at this early stage [45].

Finally, for both autologous and allogeneic therapies, as discussed with respect to clinical trials, relatively low-risk final stage or point of care manufacturing steps may be required. Under the current EU GMP framework, these manufacturing steps for an ATMP are required to be covered under a full manufacturing licence. However, where these steps are well controlled and low risk, such as a final cell expansion and medium exchange step in a closed device, a case can be made for an alternative approach such as satellite licensing under a main licence holder. This would stimulate manufacturing innovation in this area as well as facilitating multiple site clinical trials and future commercial supply where there would otherwise be a need for large numbers of manufacturing licenses to be in place for these relatively simple steps.

## 6. Reimbursement of cell therapies

Cell therapies, like other medicines, require reimbursement in order to become broadly available to patients at the end of the development process and similar to the considerations for clinical trial, manufacturing and meeting regulatory standards, early planning for reimbursement is essential. The principles and frameworks that drive reimbursement decisions for other innovative therapies apply equally to novel cell therapies. Reimbursement for cell therapies is subject to value-based assessments and demonstration of their added-value over existing therapeutic alternatives (standard of care; best supportive care). By quantifying and monetizing the magnitude of the added-value, the therapy's reimbursed price potential is determined [79]. Therefore, value-based assessments provide the link between therapy benefits (for the patient and the healthcare system) and the willingness to pay and adopt.

Core to these assessments is the availability of comparative clinical data. Direct head-to-head comparisons are the gold-standard for the purpose of health technology assessments (HTAs). However, as noted above, this can be challenging for some of the technologies discussed in this paper and the acceptability of indirect comparisons is increasing over time, especially where patient recruitment and ethical considerations present challenges with the inclusion of comparator arms in clinical trials [80]. Furthermore, generation of comparative evidence may also necessitate in-depth analysis of the clinical and economic outcomes associated with the standard of care, if this evidence is not well documented in the public domain.

Cell therapies are renowned for their high manufacturing costs which dictate a high target price in order to be commercially viable. To maximize likelihood of being reimbursed it is important to ensure that the incremental benefit novel cell therapies deliver is proportionate to their incremental cost above current therapeutic approaches. Therefore, populations of high unmet need are best targeted. Furthermore, targeting small populations can help minimize budget impact concerns and imposition of reimbursement restrictions, especially at local level where therapy uptake is often impacted by annual budgets and affordability. Therefore, when clinical development is being pursued for a larger population, *a priori* subpopulation analysis should be considered.

Another distinct feature of many cell therapies is that their incremental benefit claims extend over a longer horizon than their supporting clinical trial data at launch. This is likely to be the case across a range of technology classes where cell replacement or long-term gene modification is targeted and is the case, for example, with the approved *in vivo* gene therapy Glybera [58]. In HTA, extrapolation is commonly used to estimate measures of treatment effectiveness beyond the clinical trial period. Such measures are incorporated into health economic models, which can in turn be used to estimate lifetime costs and health outcomes. Extrapolation methods include the development of multiple parametric and semi-parametric models which are subsequently validated on the grounds of statistical considerations and clinical expert opinion on biological plausibility. Careful clinical development planning can help optimize the evidence base for extrapolations, e.g. through the use of hard rather than surrogate outcomes. However, extrapolations are always associated with uncertainty which is proportionate to the length of the extrapolation; therefore deterministic, probabilistic and structural sensitivity analysis is required to assess impact on the value claims. Furthermore, risk-sharing schemes between the manufacturers and the healthcare systems can help mitigate such uncertainty. In combination with real-world evidence planning, risk-sharing schemes [81] can provide a vehicle for rewarding the full benefits of cell therapies without overly increasing risk and financial exposure for payers. They could also provide an attractive solution to the more fragmented healthcare systems (e.g. in the USA where the healthcare provider often changes over a patient's lifetime), by only rewarding benefits as they accrue. However, such schemes necessitate regular patient follow-up and are often associated with significant clinical and administrative burden which has limited their implementation. Therefore, manufacturers should consider whether they wish to take a share of this burden in return for a scheme that could better reward long-term benefits.

The criteria applied by key market access stakeholders on deciding about the reimbursement of a novel cell therapy, vary by the features of a cell therapy and by geography. The following therapy features have an impact on how cell therapies are assessed and funded [82]:

— *Regulatory status:* There is variation in the route to market access and the reimbursement assessments applicable across different regulatory categories (ATMPs with marketing authorizations; unlicensed ATMPs under early access, temporary authorization or special availability schemes such as hospital exemptions and specials; minimally manipulated cell therapies for homologous use).
— *Size of target patient population:* Depending on size of target population, funding routes may vary from individual funding requests at local hospital level (e.g. for cell therapies targeting diseases of very low incidence/prevalence), to formal product evaluations at national and/or regional level (when larger patient populations are concerned). Furthermore, smaller target patient populations are associated with lower budget impact and, therefore, higher willingness to pay, especially in context with populations of high disease burden. This is well exemplified by the reimbursement restrictions imposed on proprietary biologics in autoimmune disease such as rheumatoid arthritis across the major European healthcare systems; such restrictions have narrowed use to refractory patients failing lower cost therapeutic options.
— *Magnitude of incremental benefit claims:* For poorly differentiated therapies many reimbursement systems enforce competitor-based pricing (e.g. reference pricing groups operating in multiple European countries). The greater the incremental benefit claims, the more likely a novel therapy will not be subject to reference pricing and existing pricing benchmarks.
— *Setting of care:* The vast majority of cell therapies in development today are expected to be hospital-only products. Their high cost requires supplementary funding arrangements outside the existing diagnosis-related group (DRG) tariffs used for hospital financing. Novel cell therapies relying on intricate interventional procedures are likely to be restricted to centres of excellence only. Where novel interventional procedures are required to deliver a cell therapy, these may need to undergo separate and prior formal assessment to that of the cell therapy itself (e.g. in England an Interventional Procedure Guidance issued by NICE (National Institute for Health and Care Excellence) would precede a Technology Appraisal (TA) if the technology is being delivered to the body in a novel way). Most importantly, the reimbursed price potential of a novel cell therapy is impacted by the cost of associated interventional procedures.
— *Impact on service delivery:* Autologous therapies in particular present additional challenges for hospital resourcing and financing as they have the potential to disrupt existing treatment algorithms by introducing additional steps (e.g. bone marrow aspiration); therefore, assessments of such therapies can demand additional considerations including reallocation of healthcare resources and re-engineering of existing service delivery processes.

Geography is another variable that impacts applicable methodology to reimbursement assessments. There is variation across countries and regions in the relative importance of clinical and economic considerations and the type of health economics frameworks applied (e.g. cost-effectiveness, cost-utility, cost-consequence, efficiency frontier, budget impact). Furthermore, certain countries operate international price referencing mechanisms in determining the reimbursed price potential for novel therapies [83].

For high cost cell therapies with clear benefits for the patient and the healthcare system, the use of health economics in substantiating reimbursed price potential can help them escape existing pricing benchmarks and access rewards proportionate to the full benefits they deliver.

In the UK, bodies such as NICE and SMC (Scottish Medicines Consortium) undertake HTAs that leverage clinical-effectiveness and cost-effectiveness considerations. Whereas NICE undertakes a variety of assessments in order to make recommendations on the use of new and existing therapies within the NHS, only two types of its assessments result in binding obligations for NHS commissioning: the TA and the Highly Specialised Technology Evaluations (HSTE). The latter is for therapies with patient populations small enough so that treatment is concentrated in very few centres in the NHS, whereas the former is for therapies targeting larger patient populations [84]. The assessment methodology applied in NICE TA is that of cost-utility [84], i.e. a cost-effectiveness analysis in which effectiveness is measured in terms of Quality-Adjusted Life Years (QALYs). By comparing the incremental costs of introducing a new treatment to the incremental benefits (QALYs) it delivers over the standard of care, an Incremental Cost-Effectiveness Ratio (ICER) is calculated. ICER values below £30 000 are associated with favourable NICE decisions for NHS adoption of new treatments. Unlike NICE TA, NICE HSTE does not use clearly defined ICER thresholds to support its recommendations.

Similar to NICE TA assessment frameworks leveraging cost-effectiveness operate in Canada, Netherlands, Sweden, Australia and have recently been introduced in France for innovative therapies; however, there is variation in the size and application of the ICER thresholds across these countries [85].

**Figure 4.**
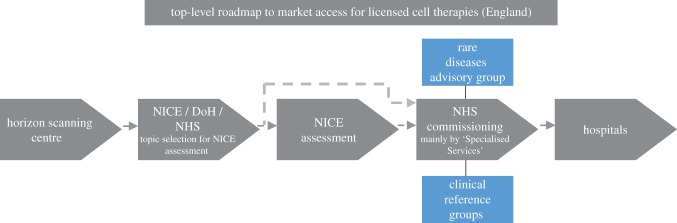
Flowchart for NHS adoption of licensed cell therapies in England. Multiple market access stakeholders are involved in determining NHS adoption. The relative importance of these stakeholders varies by type of cell therapy.

Figure 4 presents the route to NHS adoption for licensed cell therapies in England diagrammatically [82]. Following notification from the Horizon Scanning Centre on therapies likely to pursue NHS adoption, NICE, the Department of Health (DoH) and the National Health Service (NHS), apply a set of defined and transparent selection, elimination and prioritization criteria to determine which therapies are most relevant for TA and HSTE assessments. Unlicensed and poorly differentiated treatments are eliminated. High cost therapies targeting small populations (less than 500 in England) with chronic and severely disabling conditions of high unmet need are likely to be channelled through HSTE, however, the requirement for the therapy to be delivered on chronic basis needs to be met [86]. Therefore, for cell therapies targeting small patient populations and being administered for a finite period, no NICE assessment exists that results in binding obligations for the NHS.

For cell therapies that have not undergone NICE TA or HSTE, the commissioning decision in England lies entirely with NHS England and more specifically with the NHS Specialised Services with input from the Clinical Reference Groups; for therapies targeting rare diseases, the Rare Diseases Advisory Group is also consulted [82]. Where non-binding types of NICE guidance are available (e.g. Interventional Procedures Guidance) these can help inform the decision-making of the NHS commissioners but without the obligation to be implemented.

In Europe, at the time of writing, except for ChondroCelect that had secured reimbursement in the Netherlands, Belgium and Spain, in other territories and for all other licensed ATMPs (i.e. MACI, Glybera, Provenge, Holoclar), reimbursement assessments were in progress.

## 7. Conclusion

The future development of cell therapies is increasingly focusing not just on the translational space and addressing the challenges of proceeding through clinical development but increasingly also on strategies that will lead to successful commercialization. Pathfinder therapies, including the five currently approved ATMPs in the EU, demonstrate that successful marketing authorizations can be secured and also exemplify the importance of enabling developments such that the number of approved therapies and speed of their development to meet the needs of patients can be increased.

The classification system of cell therapies based on their underlying technology groups proposed in this paper shows how common themes can be found across apparently diverse groups of therapies. These technologies are at different stages of development and adoption, with some, such as genome editing still at the pre-clinical stage but likely to advance very quickly based on the advances made in the areas of *in vivo* and *in vitro* gene modification as well as cell reprogramming.

The clinical trial, manufacturing and regulation of the different classes of cell-technology exemplify both how principles and learnings from existing medicines, both small molecule based and biologic, can be applied to the cell-therapy class but also where there are important differences because of the nature of the cells themselves and their inherently variable properties.

The future pricing and reimbursement potential of a novel cell therapy is another critical parameter to be factored in from early in the development process. Willingness to pay and therapy adoption depend on the magnitude of the novel therapy's incremental benefits over existing therapeutic alternatives. This means that manufacturers need to ensure that clinical and economic comparative evidence is generated during clinical development wherever possible in order to support negotiations at launch with key market access stakeholders and additionally, innovative mechanisms are also emerging to reflect the novel characteristics of these therapies, their benefits and uncertainties.

Overall, the rapid scientific advancement in this area and emerging examples of cell-based medicines which are transformative for patient care will continue to drive progress, translation and ultimately commercialization.
